# An automated algorithm for the detection of cortical interruptions on high resolution peripheral quantitative computed tomography images of finger joints

**DOI:** 10.1371/journal.pone.0175829

**Published:** 2017-04-20

**Authors:** M. Peters, A. Scharmga, J. de Jong, A. van Tubergen, P. Geusens, J. J. Arts, D. Loeffen, R. Weijers, B. van Rietbergen, J. van den Bergh

**Affiliations:** 1 Department of Internal Medicine, Division of Rheumatology, Maastricht University Medical Centre, Maastricht, the Netherlands; 2 CAPHRI, School for Public Health and Primary Care, Maastricht University, Maastricht, the Netherlands; 3 NUTRIM School for Nutrition and Translational Research in Metabolism, Maastricht University, Maastricht, the Netherlands; 4 Department of Radiology, Maastricht University Medical Centre, Maastricht, the Netherlands; 5 Faculty of Medicine and Life Sciences, Hasselt University, Hasselt, Belgium; 6 Department of Orthopaedic Surgery, Maastricht University Medical Centre, Maastricht, the Netherlands; 7 Faculty of Biomedical Engineering, Eindhoven University of Technology, Eindhoven, the Netherlands; 8 Department of Internal Medicine, VieCuri Medical Centre, Venlo, the Netherlands; Vanderbilt University, UNITED STATES

## Abstract

**Objectives:**

To introduce a fully-automated algorithm for the detection of small cortical interruptions (≥0.246mm in diameter) on high resolution peripheral quantitative computed tomography (HR-pQCT) images, and to investigate the additional value of manual correction of the automatically obtained contours (semi-automated procedure).

**Methods:**

Ten metacarpophalangeal joints from seven patients with rheumatoid arthritis (RA) and three healthy controls were imaged with HR-pQCT. The images were evaluated by an algorithm according to the fully- and semi-automated procedure for the number and surface of interruptions per joint. Reliability between the fully- and semi-automated procedure and between two independent operators was tested using intra-class correlation coefficient (ICC) and the proportion of matching interruptions. Validity of single interruptions detected was tested by comparing it to visual scoring, as gold standard. The positive predictive value (PPV) and sensitivity were calculated.

**Results:**

The median number of interruptions per joint was 14 (range 2 to 59) and did not significantly differ between the fully- and semi-automated procedure (p = 0.37). The median interruption surface per joint was significantly higher with the fully- vs. semi-automated procedure (respectively, 8.6mm^2^ vs. 5.8mm^2^ and 6.1mm^2^, p = 0.01). Reliability was almost perfect between the fully- and semi-automated procedure for both the number and surface of interruptions (ICC≥0.95) and the proportion of matching interruptions was high (≥76%). Also the inter-operator reliability was almost perfect (ICC≥0.97, proportion of matching interruptions 92%). The PPV ranged from 27.6% to 29.9%, and sensitivity from 69.7% to 76.3%. Most interruptions detected with the algorithm, did show an interruption on a 2D grayscale image. However, this interruption did not meet the criteria of an interruption with visual scoring.

**Conclusion:**

The algorithm for HR-pQCT images detects cortical interruptions, and its interruption surface. Reliability and validity was comparable for the fully- and semi-automated procedures. However, we advise the use of the semi-automated procedure to assure quality. The algorithm is a promising tool for a sensitive and objective assessment of cortical interruptions in finger joints assessed by HR-pQCT.

## Introduction

Peri-articular cortical interruptions are one of the characteristic features of bone involvement in rheumatoid arthritis (RA) [1,2]. They may occur already at an early stage and are predictors of further radiographic progression [3]. The presence, number, and size of interruptions within a joint and the number of joints affected are associated with a poor functional outcome [2,4,5].

Conventional radiographs (CR) are considered the gold standard for detection of cortical interruptions in hand joints in RA. However, CR has its limitations since the sensitivity of the detection of cortical interruptions is low compared with other imaging modalities such as Computed Tomography (CT), magnetic resonance imaging (MRI) and Ultrasound (US) [6–9]. High resolution peripheral quantitative CT (HR-pQCT) is a low-dose imaging technique that is able to assess the three-dimensional (3D) bone structure of the peripheral skeleton at the micro-scale (82μm isotropic voxel size) in vivo. Studies with HR-pQCT in finger joints showed higher sensitivity for the detection of cortical interruptions compared with US, CR and MRI [10–13].

Cortical interruptions, together with changes in the underlying trabecular bone architecture, such as absence of trabeculae, with or without surrounding sclerosis, are a hallmark of bone erosions. Several studies reported results on the visual inspection of the presence, number and size of cortical interruptions as part of analyzing erosions using HR-pQCT [10–20]. High intra- and inter-rater reliability for the visual scoring of the number of erosions was found, with intra-class correlation coefficients (ICC) and kappa values ranging from 0.6 to 1.0 [10–12,18]. However, in all studies only established erosions and thus relatively large cortical interruptions were scored (mean width >2mm [15,21], mean depth >2mm [15,21], mean maximal dimension >3mm [11] and mean volume >4mm^3^ [14,19]). Studies that systematically investigate the visual detection of smaller cortical interruptions on HR-pQCT images are currently lacking, but it is known that this is a laborious, complex and difficult process [22]. Although these small interruptions might not be specific for RA [10], they might be prone to bone resorption early in the course of RA and can therefore be the first signs of erosions [23]. Moreover, it has been shown that the number of these small interruptions is increased in patients with more active disease, suggesting a link to synovitis [10].

Automated algorithms are an alternative to visual scoring of cortical interruptions on HR-pQCT images. Recently, an algorithm has been developed that accurately measured erosion volume and erosion surface area, but still required an operator to visually identify the erosion and manually set a seeding point in the erosion [19]. A fully-automated detection method of (small) cortical interruptions was not yet available.

Here, we introduce a newly developed, fully-automated algorithm for the detection of small cortical interruptions (≥0.246mm in diameter) on HR-pQCT images. Because the algorithm might be susceptible for errors in the contouring, we investigated the additional value of correction of the contours by an operator (semi-automated procedure) as suggested by Burghardt et al. [24]. In addition, we tested the validity of the cortical interruptions detected by the algorithm compared to visual scoring, as gold standard.

## Materials and methods

### Patients

We selected a convenient sample of seven patients with RA (mean age 53.4 (SD 7.3) years, mean disease duration 8.2 (SD 9.8) years) and three healthy controls (HCs) (mean age 48.0 (SD 7.8) years) from the observational MOSA-Hand cohort study, including 41 patients with RA and 38 HCs at the Maastricht University Medical Center, the Netherlands. The subjects were considered eligible when the HR-pQCT scan was of sufficient quality (≤ grade 3, as described by Pialat et al. [25]), and the joint was not destroyed. All patients with RA fulfilled the 2010 American College of Rheumatology (ACR)/European League Against Rheumatism (EULAR) classification criteria for RA [26]. None of the HCs suffered from hand joint complaints. Ethical approval was obtained from the ethics board of the academic hospital Maastricht, the Netherlands (NL42300.068.12 / METC 12-2-037). All participants signed informed consent.

### HR-pQCT scanning procedure

MCP joints of both hands in patients with RA and the dominant hand in HCs were scanned with HR-pQCT (XtremeCT1, Scanco Medical AG, Switzerland). The scanning was performed at clinical in vivo settings, i.e. at 60kVp tube voltage, 900μA tube current, 100ms integration time and 82μm isotropic voxel size. The reference line was placed on top of the second metacarpal head, such that the scan region covered a length of 16.56mm in proximal direction and 10.50mm in distal direction (total scan length 27.06mm, 330slices). The total scanning time was approximately 9minutes and the effective dose was <9μSv. In each patient, the third MCP joint of the first scan that was available of a patient (either left or right sided) was selected for this study.

### Cortical interruption detection algorithm

**Fig 1:**
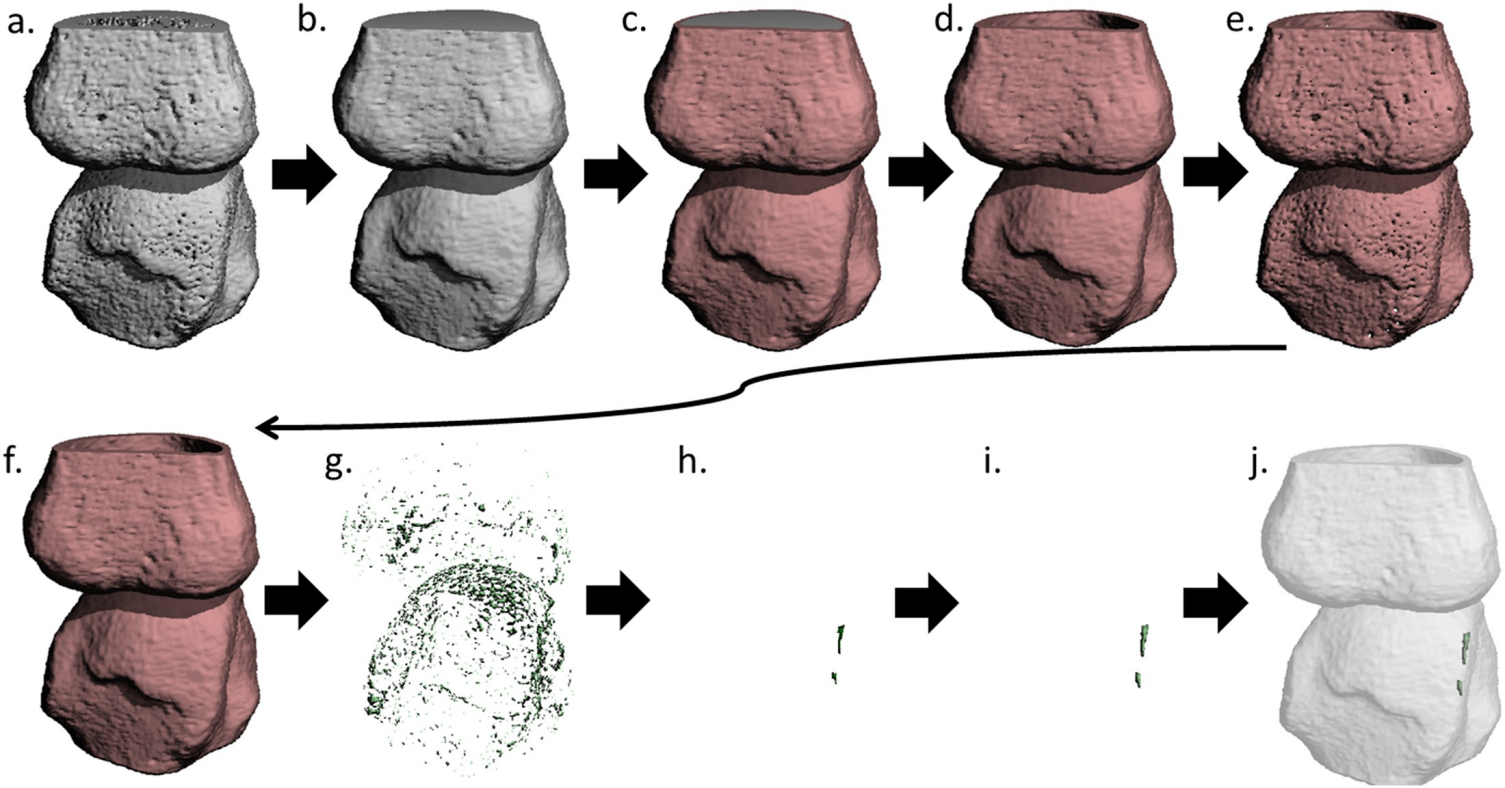
Representation of the steps executed by the algorithm. Representation of the steps executed by the algorithm applied to a 3D reconstruction of a HR-pQCT image of an MCP joint. Based on the outer margin contour of the original bone structure (a.) a solid volume is created (b.). The outer shell of this volume is segmented and depicted in red (c.) using an erosion operation. This is the cortical mask (d.), which is used to identify the cortical bone (e.). This cortical bone is dilated to fill small cavities (f.). Next, the image is inverted (g.) and only interruptions that were connected to the endosteal and periosteal boundary are selected (h.). The remaining cortical interruptions are dilated to their original volume (i.) and the results can be visually inspected by adding a transparent cortical mask (j.).

**Fig 2:**
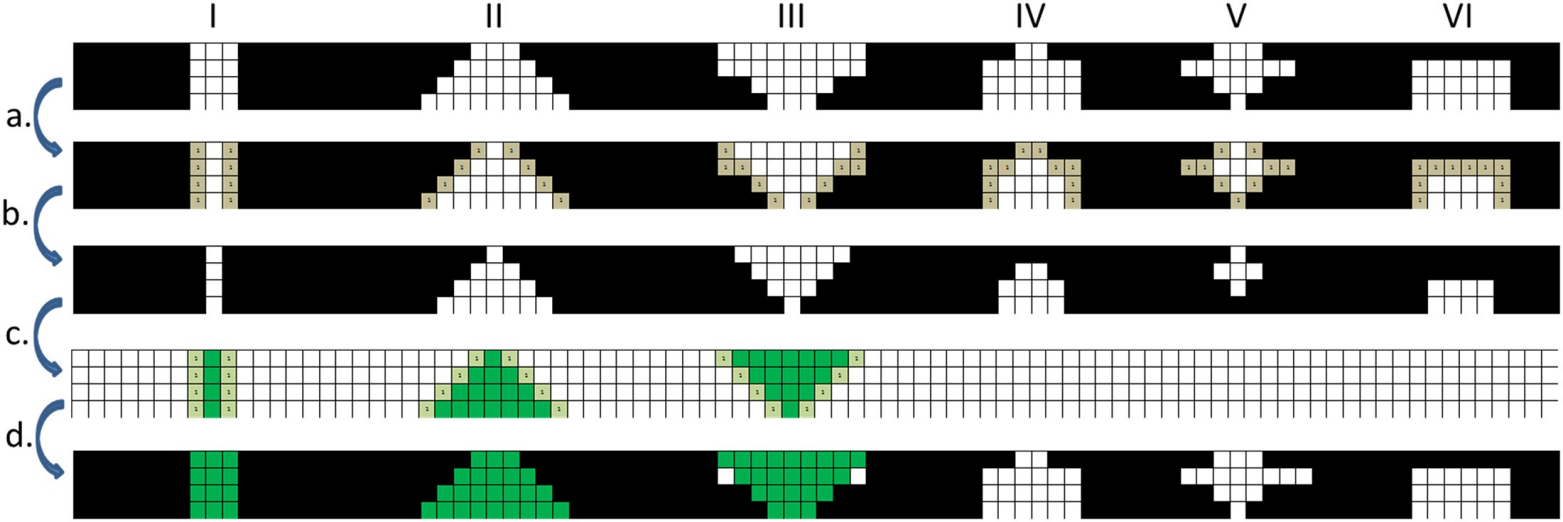
2D examples of cortical interruptions on voxel level that are detected and are not detected by the algorithm. 2D examples of cortical interruptions on voxel level that are detected (I-III) and are not detected (IV-VI) by the algorithm. The following steps are made by the algorithm: The original cortex (step a, depicted in black) is dilated with 1 voxel (step b, depicted in grey). The cortex is then inverted (step c), only interruptions that are connected with the endosteal and periosteal boundary are selected and dilated to (approximately) its original size. The interruptions that are detected are subsequently displayed in the cortex (green) (d). Interruptions that are not connected to both the endosteal and periosteal boundary (IV-VI) with at least 3 pixels are not detected by the algorithm.

We developed the automated algorithm in several steps. In order to reduce the influence of noise on the images, we considered discontinuities in the cortex as cortical interruptions only if there was an opening of at least 5 voxels through the cortex and connected to both the periosteal and endosteal boundary of the cortical mask. This corresponds to a discontinuity in at least 3 consecutive slices and a width of at least 3 voxels (= 0.246mm). In addition, a cortical mask with a constant thickness of 4 voxels (= 0.328mm) was chosen because this approached the average cortical thickness in the MCP joints in this study, which was 0.39mm. We decided to set the thickness of the cortical mask lower, because the thickness of the cortex at the rim of the joint, where we expected most cortical interruptions, is in general thinner [14]. The minimal intra-cortical interruption volume that can be detected by our algorithm is 20 voxels (0.011mm^3^), based on an opening of at least 5 voxels * depth of 4 voxels. The 4 steps of the algorithm are explained below and displayed in Figs 1 and 2 and S1 Fig.

**First**, the outer contour of the cortex is identified. We applied a modified version of the auto-contouring algorithm developed for periosteal segmentation of the distal radius and tibia [24,27]. Modifications on this algorithm were made for the structure approximation of the bone, which was performed using Gaussian filtering (sigma = 0.8, support = 1 voxel) with a constant threshold of 105 instead of 120 per 1000 of the maximum possible voxel value, because the cortex in finger joint is thinner and lower mineralized compared to radius and tibia in order to reduce the effect of partial volumes. In addition, and in contrast, with the originally developed algorithm, 7 instead of 10 closing steps (dilation followed by erosion in 3D) were performed, because finger joints are smaller compared to radius and tibia. This algorithm is explained in online Fig 1.

**Second**, a binary 3D model is created by extracting the voxels that contain mineralized bone using the standard evaluation protocol from the manufacturer for radii and tibia, which includes Laplace-Hamming filtering and thresholding [28]. An example of a binary 3D model of an MCP joint is shown in Fig 1a.

**Third**, a cortical mask is generated by creating a 3D binary solid volume based on the outer margin contour (Fig 1b). This volume is then eroded in 3D by a distance of 4 voxels. The eroded volume is subtracted from its original volume, resulting in the outer shell of the contoured volume with a constant thickness of 4 voxels (Fig 1c). This shell is used as the mask (Fig 1d) to identify the cortical bone (Fig 1e).

**Fourth**, the cortical bone is analyzed for discontinuities that meet the preset criteria of a cortical interruption (i.e. a connectivity of 5 voxels through the cortex and to both the periosteal and endosteal boundary of the cortical mask). First, the bone within the cortical mask is dilated in 3D by a distance of 1 voxel (Fig 1f). Second, the image is inverted (Fig 1g), and only interruptions that remain connected to both the endosteal and periosteal boundaries of the cortical mask are selected for further image processing (Fig 1h). These interruptions are dilated again in 3D by 1 voxel to restore the original volume (Fig 1i) and are considered cortical interruptions. Based on this analysis, an image can be created with a transparent cortical mask as reference (Fig 1j). Subsequently, the total number of cortical interruptions and the intra-cortical volume of each cortical interruption is determined using a function that identifies unconnected structural components and their volume. The interruption surface is calculated by dividing the intra-cortical interruption volume by the thickness of the cortical mask (= 0.328mm). Fig 2 shows 2D examples of cortical interruptions on voxel level, which are detected and not detected by the algorithm. Only interruptions on at least 3 consecutive slices and a width of at least 3 voxels through the cortex are detected. The algorithm is enclosed as supplementary file (S1 File).

### Correction of the contours

All HR-pQCT images were also evaluated by the semi-automated procedure in which the contour generated in step 1 of the fully-automated procedure is visually inspected and, if necessary, corrected by an operator as recommended by Burghardt et al. [24]. To test the inter-operator reliability, this correction was performed by two operators independently.

### Visual scoring

All HR-pQCT images were scored visually on cortical interruptions for comparison with the algorithm. A cortical interruption was defined as a clear interruption of the cortex, seen on two consecutive slices on two orthogonal planes (on transverse, and on sagittal or coronal plane) [29,30].

### Statistical analysis

In this study, the results of the fully-automated procedure (AUTO), the two operators (OP1 and OP2) of the semi-automated procedure, and visual scoring were compared. Descriptives of the number of interruptions and the interruption surface were calculated. Friedman test was used for comparison across the operators and visual scoring. Post-hoc analyses were performed using Wilcoxon signed-rank test between the operators, and between the operators and visual scoring.

Reliability between AUTO and the semi-automated procedure, and between OP1 and OP2 for the number of interruptions and interruption surface on the joint level was estimated by ICCs with a two-way random model and absolute agreement. In addition, the reliability on the joint level between the algorithm (AUTO, OP1 and OP2) and visual scoring for the number of interruptions was obtained using ICCs.

Reliability was rated according to Landis et al.: <0.00 poor, 0.00–0.20 slight, 0.21–0.40 fair, 0.41–0.60 moderate, 0.61–0.80 substantial, 0.81–1.00 almost perfect [31]. In addition, reliability was evaluated by the proportion of matching interruptions across the operators. Interruptions were counted as matching interruptions if they overlapped with at least 20 voxels (0.011mm^3^), because this is the smallest intra-cortical interruption volume that could be detected with this algorithm. The proportion of matching interruptions was calculated for the presence (yes/no) of a cortical interruption on exactly the same location according to Eq 1. Moreover, the validity of the algorithm to detect single interruptions on exactly the same location was evaluated by calculation of the positive predictive value (PPV, Eq 2), and sensitivity (Eq 3) using the visual scoring as gold standard. Statistical analysis was performed using IBM SPSS Statistics for Windows, Version 20.0 (IBM Corp., Armonk, NY).

$$Proportion\;of\;matching\;interruptions=\;\frac{\begin{array}{c} nr.\;of\;matching\;interruptions_{OP12} \end{array}}{\begin{array}{c} nr.\;of\;interruptions_{OP1}+nr.\;of\;interruptions_{OP2}-nr.\;of\;matching\;interruptions_{OP12} \end{array}}*100\%$$ (1)

Nr. of interruptions_OP1_: Number of interruptions detected by 1^st^ operator

Nr. of interruptions_OP2_: Number of interruptions detected by 2^nd^ operator

Nr. of matching interruptions_OP12_: Number of matching interruptions between 1^st^ and 2^nd^ operator

$$\text{PPV}=\;\frac{\begin{array}{c} \text{nr}.\text{ of matching interruptions} \end{array}}{\begin{array}{c} \text{nr}{\text{. of interruptions}}_{\text{algorithm}} \end{array}}*100\text{\%}$$ (2)

PPV: Positive predictive value

Nr. of interruptions_algorithm_: Number of interruptions detected by the algorithm

Nr. of matching interruptions: Number of matching interruptions between the algorithm and visual scoring

$$\text{Sensitivity}=\;\frac{\begin{array}{c} \text{nr}.\text{ of matching interruptions} \end{array}}{\begin{array}{c} \text{nr}{\text{. of interruptions}}_{\text{visual scoring}} \end{array}}*100\text{\%}$$ (3)

Nr. of interruptions_visual scoring_: Number of interruptions detected with visual scoring

Nr. of matching interruptions: Number of matching interruptions between the algorithm and visual scoring

## Results

### Visual impression

#### Output algorithm

**Fig 3:**
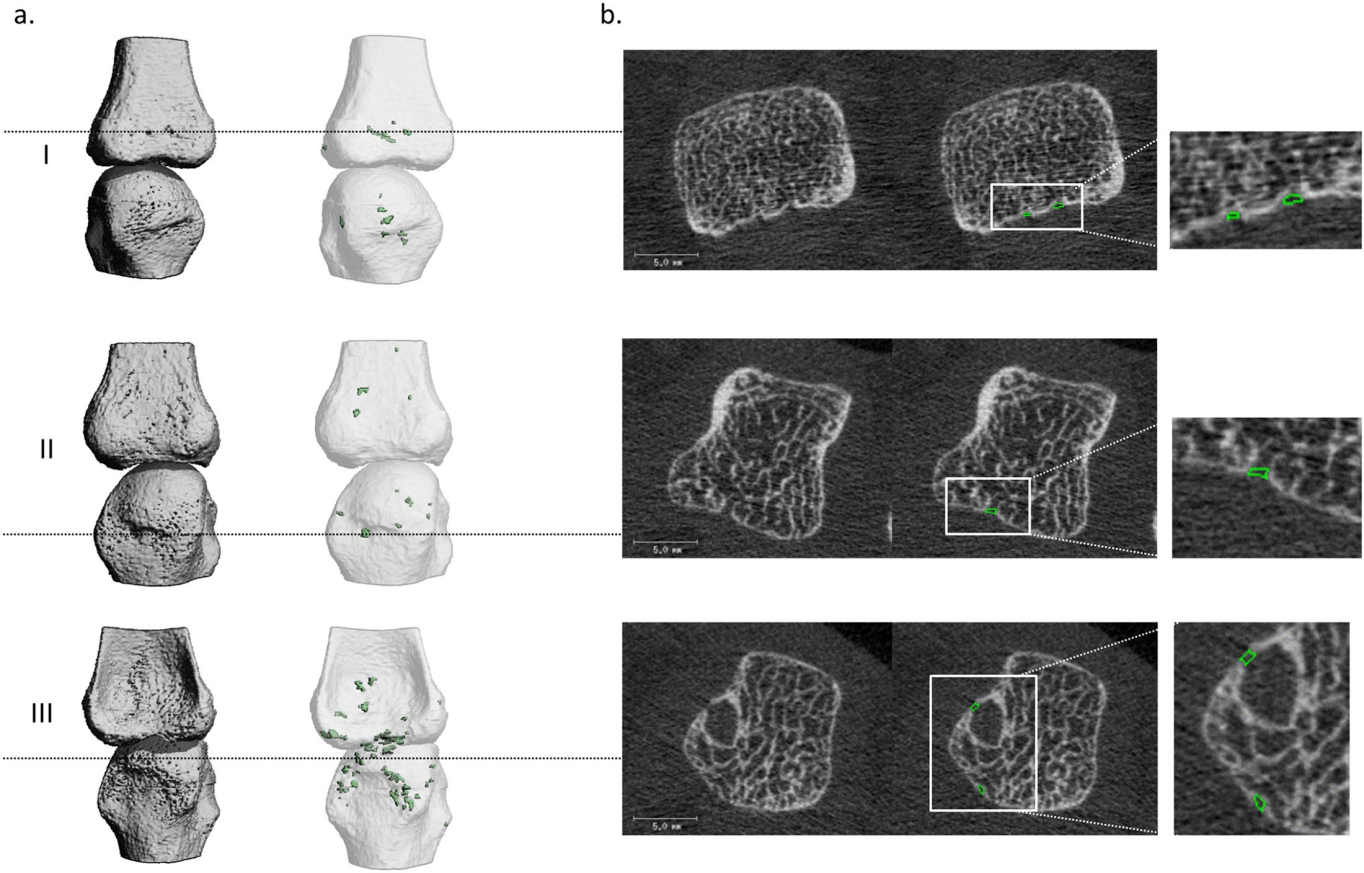
Examples of the 2D and 3D outputs of the algorithm. Typical examples of a 3D reconstruction of an MCP joint for a HC (I), a patient with early RA (<2 years since diagnosis) (II), and a patient with late RA (>10 years since diagnosis) (III), with the corresponding 3D outputs of the algorithm. The cortical region is indicated in transparent white. Cortical interruptions of ≥0.246mm that are detected by the algorithm are shown in green. b) Corresponding 2D grayscale images with in green cortical interruptions detected by the algorithm.

Both the fully- and semi-automated procedures detected multiple cortical interruptions in HCs and patients with RA (Fig 3). Fig 3 shows a typical example of 3D reconstructions of an MCP joint of an HC (I), a patient with early RA (<2 years since diagnosis) (II), and a patient with late RA (>10 years since diagnosis) (III), and the detected interruptions of the fully-automated procedure. Multiple cortical interruptions (depicted in green) can be seen in the 3D images and most interruptions are located at the rim of the joint (Fig 3a). Fig 3b shows corresponding 2D grayscale images of examples of interruptions that were detected by the fully-automated as well as the semi-automated procedure.

#### Correction of the contours

**Fig 4:**
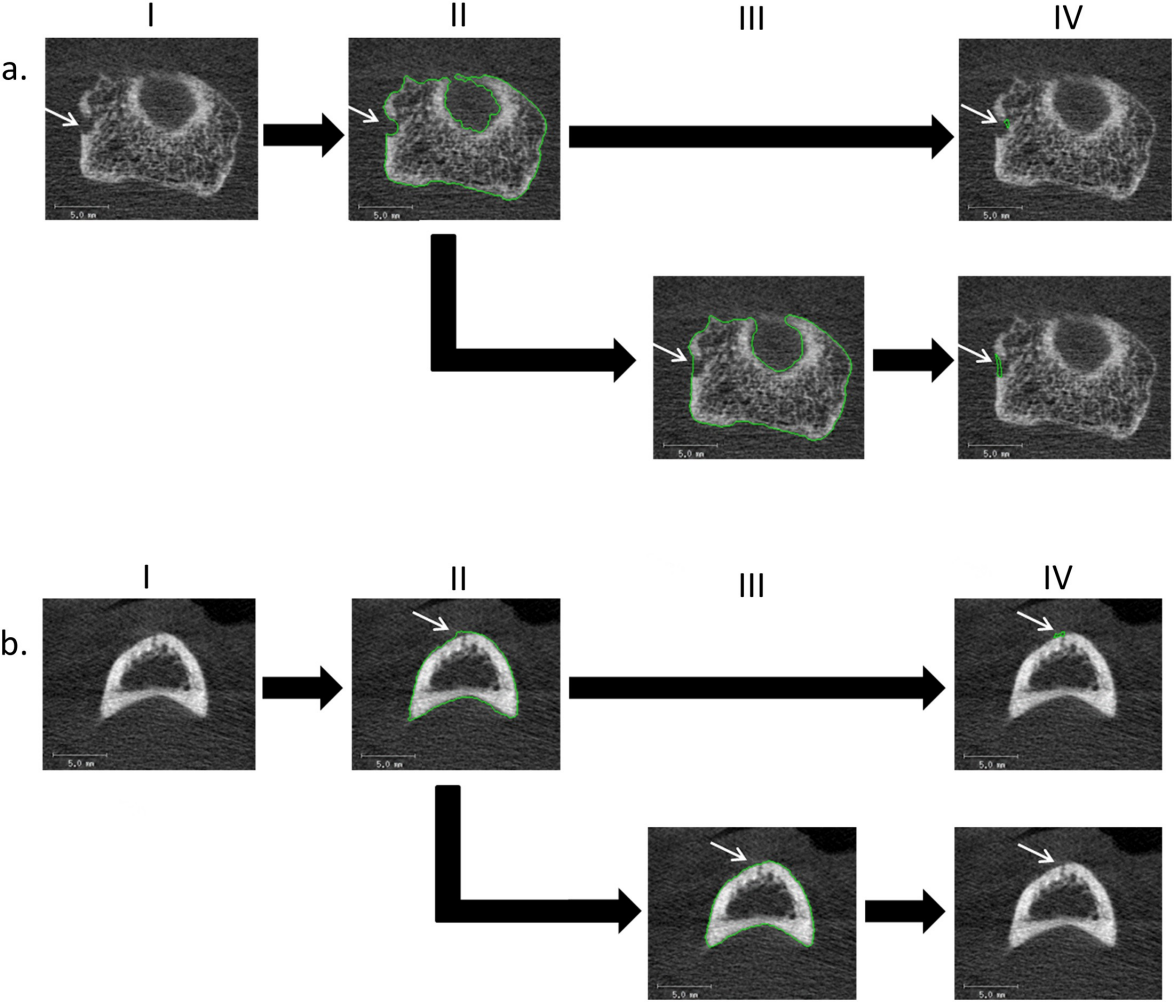
Examples of contours that were manually corrected. Typical examples of 2D grayscale images in which the contour is manually corrected by the operators. In a) a large cortical interruption is shown (a. I, arrow). The automatically obtained contour does not follow the outer margin of the original structure at the cortical interruption (a. II). The operators therefore corrected the contour (a. III) to accurately detect the large cortical interruption (IV). In b) a small motion artifact is shown (I). Due to this motion artifact, the automatically obtained contour was not tight around its original structure (II). The operators corrected this (III) and therefore no interruption was detected (IV).

Fig 4 shows examples of contours obtained with the fully-automated procedure that were manually corrected by the operators. In two cases, large cortical interruptions were corrected by both operators. For small cortical interruptions (diameter<1mm) no correction was necessary by the operators. Fig 4a.I shows an example of a large cortical interruption (diameter>1.0mm, white arrow). The contour obtained with the fully-automated procedure did not follow the outer margin of the original structure at the location of the cortical interruption (Fig 4a.II). Both operators corrected the contour (Fig 4a.III, white arrow) to more precisely detect the size of the large cortical interruption (Fig 4a.IV).

Fig 4b.I shows an example of a small motion artifact (white arrow). Due to this motion artifact, the contour obtained with the fully-automated procedure was not tight around the original structure (Fig 4b.II), which led to a false detection of an interruption (Fig 4b.IV). Both operators corrected the contour (Fig 4b.IV).

### Quantitative comparison

Table 1 shows the number of interruptions and interruption surface detected by the algorithm (AUTO, OP1 and OP2), and visual scoring. The median number of interruptions was 14 and ranged from 2 to 59 interruptions per joint for the algorithm when including all operators. The median number of interruptions with visual scoring was 5 and ranged from 3 to 22 interruptions per joint. Statistical significant differences were found across the algorithm and visual scoring for the number of interruptions (p<0.01). The post-hoc analysis showed significant differences between the algorithm (AUTO, OP1 and OP2) versus visual scoring (all, p≤0.01).

**Table 1:** The number of cortical interruptions and interruption surface per joint detected by the algorithm and with visual scoring.

	Algorithm			Visual	
	AUTO	OP1	OP2		p-value *
**Number of Interruptions**	15.0^$^ [6–49]	13.5^$^ [2–59]	14.0^$^ [3–51]	5.0^$^ [3–22]	<0.01
**Interruption surface (mm^2^)**	8.6^#^ [2.3–67.7]	5.8^#^ [1.5–68.9]	6.1 [2.6–76.7]		0.02

Values are displayed as: median [min—max],

AUTO = fully-automated procedure

OP1 = semi-automated procedure manual correction by operator 1

OP2 = semi-automated procedure manual correction by operator 2

* p-values obtained from Friedman test

^$^ p ≤0.01 obtained from post-hoc Wilcoxon signed-rank test (algorithm (AUTO, OP1 and OP2) vs. visual)

^#^ p <0.05 obtained from post-hoc Wilcoxon signed-rank test (AUTO vs. OP1)

The median of the interruption surface per joint was 7.0mm^2^ and ranged from 1.5mm^2^ to 76.7mm^2^ when including all operators. The average surface of a single interruption was <1.0mm^2^. The interruption surface per joint was significantly lower with the semi-automated vs. the fully-automated procedure (Table 1). The post-hoc analysis showed a significant difference between AUTO and OP1 (p = 0.02), but no significant difference between AUTO and OP2 (p = 0.07), and OP1 and OP2 (p = 0.29).

The reliability between AUTO and both OP1 and OP2 was almost perfect for both the total number of interruptions and the interruption surface (ICC ≥0.95, p<0.01). The majority of interruptions were detected on exactly the same location (≥76.6%) (Table 2). The inter-operator reliability was also almost perfect (ICC ≥0.97, p<0.01), as was the proportion of matching interruptions (82.0%).

**Table 2:** Reliability of the algorithm.

	AUTO vs. OP1	AUTO vs. OP2	Inter-operator
**Number of Interruptions**	ICC 0.96 (95% CI 0.87–0.99)	ICC 0.95 (95% CI 0.83–0.99)	ICC 0.97 (95% CI 0.90–0.99)
**Interruption Surface**	ICC 0.98 (95% CI 0.77–1.00)	ICC 0.96 (95% CI 0.85–0.99)	ICC 0.98 (95% CI 0.92–1.00)
**Proportion of matching Interruptions**	81.1%	76.6%	82.0%

Reliability of the fully-automated vs. semi-automated (AUTO vs. OP1 and AUTO vs. OP2) and semi-automated inter-operator (OP1 vs. OP2) of the algorithm is shown. ICCs are calculated on the total number of cortical interruptions and interruption surface in all joints. Proportion of matching interruptions is calculated on the presence of a cortical interruption (yes/no) on exactly the same location. ICC = intra-class correlation coefficient, 95%CI = 95% confidence interval

### Comparison to visual scoring

**Fig 5:**
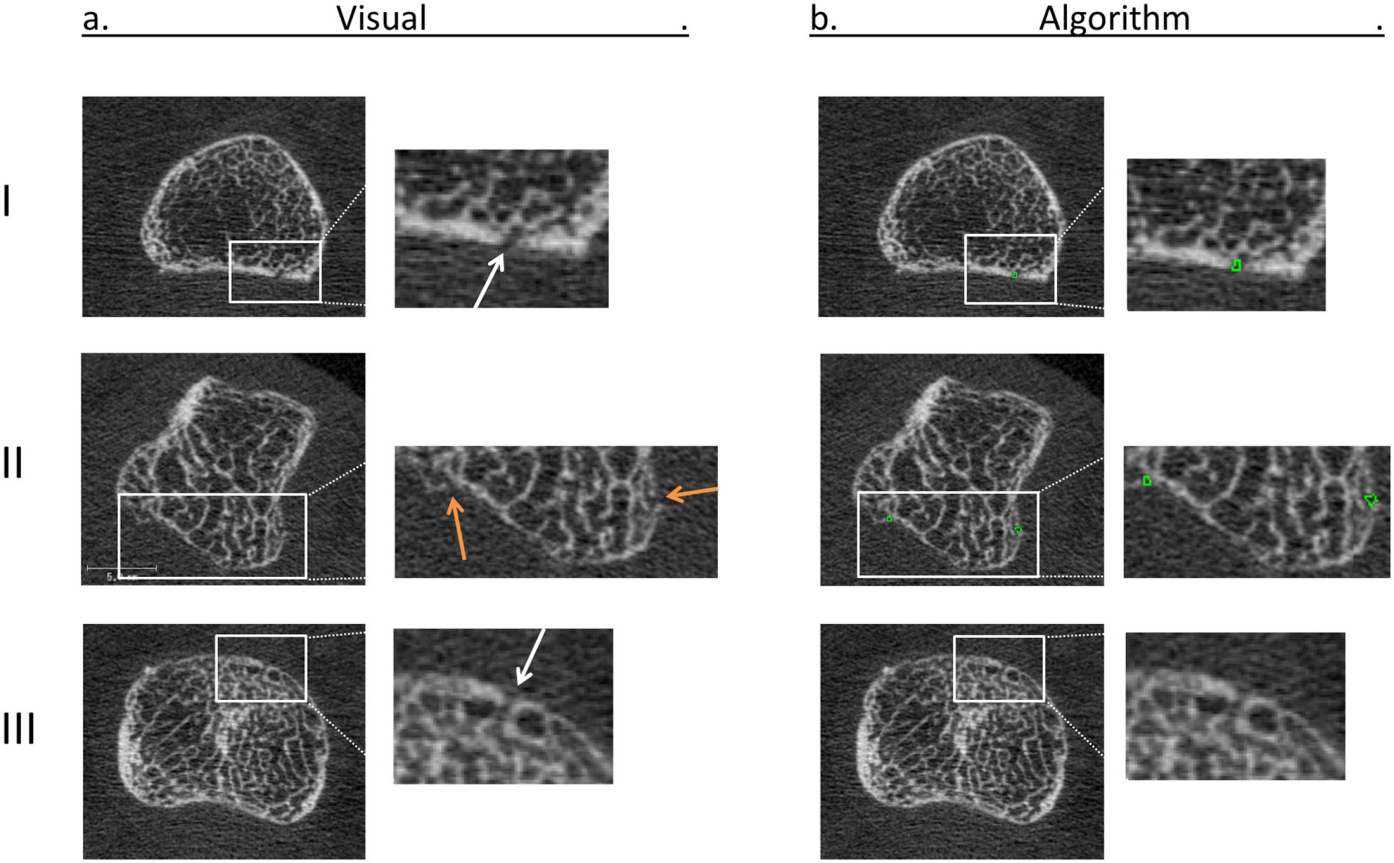
2D examples of interruptions detected visually, and with the algorithm. Typical examples of a 2D grayscale images of interruptions detected visually (a), and with the algorithm (b). (I.) Shows an interruption detected visually (white arrow) as well as with the algorithm (green circle). (II.) Shows interuptions detected by the algorithm which were not detected visually (orange arrows). Small cortical interruptions could be observed, but not meeting the criteria of a cortical interruption in the visual scoring (II.). (III.) Shows a small interruption that was detected visually (white arrow), but not with the algorithm.

The reliability between the algorithm (AUTO, OP1 and OP2), and visual scoring was fair to moderate for the number of interruptions (ICC ranging from 0.38 to 0.45). The PPV of interruptions detected by the algorithm compared to visual scoring was fair (PPV ranging from 27.6% to 29.9%). The sensitivity of the algorithm compared to visual scoring was substantial (sensitivity ranging from 69.7% to 76.3%). Examples of interruptions detected visually and with the algorithm are shown in Fig 5. An example of an interruption detected visually as well as with the algorithm is shown (Fig 5.I). Fig 5.II shows interruptions detected with the algorithm which did not meet with the criteria of visual scoring. Fig 5.III shows an interruption that was detected visually, but missed by the algorithm.

## Discussion

In this study, we introduced a fully-automated algorithm to detect small cortical interruptions in HR-pQCT images and tested the additional value of correction by an operator. In addition, we tested the validity of interruptions detected by the algorithm by comparing it to visual scoring, as gold standard. The interruption surface detected with the fully-automated procedure was significantly higher compared with the semi-automated procedure, but the absolute number of interruptions did not significantly differ. Reliability was almost perfect between both procedures (ICC ≥0.95) meaning that the manual correction of the contours was of little additional value. In addition, the proportion of matching interruptions on exactly the same location was substantial to almost perfect across the operators (≥76.6%). Lastly, the validity of a single interruption was similar for both procedures. We found that most interruptions detected by the algorithm were not identified as a cortical interruption with the visual scoring (70–72%). However, in most cases an interruption was present, but did not met the preset criteria (Fig 5.II). In addition, most interruptions detected visually were detected by the algorithm (70–76%). However, it remains important that the images are rated on motion artifacts before applying the fully-automated algorithm. Only a scan quality with grade ≤3 according to Pialat et al. [25], should be included in the analysis. Even small motion artifacts may lead to false positive detection of cortical interruptions as exemplified in this study (Fig 4b). Compared to visual scoring of the number of cortical interruptions [10–19], our approach has the advantage of providing an automated and objective measure of evaluation of cortical interruptions and its interruption surface, including small interruptions.

The number of cortical interruptions per joint detected in our study (median 14, ranging from 2 to 59) is substantially higher than observed in other studies using HR-pQCT, ranging from 0.5 to 2.9 interruptions per MCP joint [10,11,15,16,18,19,21]. This can mainly be explained by the fact that our algorithm detects smaller cortical interruptions (mean interruption surface < 1mm^2^, equivalent to a width of <1.13mm when assuming a circle) compared with a visual scoring of the number of interruptions (mean width >2mm [15,21]). Furthermore, in these studies the number of interruptions was scored on 2D grayscale images whereas our automated algorithm detects interruptions on 3D binary images, which has the advantage of having an objective manner to distinguish between bone and non-bone. The use of binary images has been validated earlier in human tibia and radius [32,33]. A potential disadvantage of binary images is the partial volume effect, which mainly affects regions where the cortical bone is thin, i.e. close to the image spatial resolution, leading to the misperception that an interruption is present in the binary image. We showed examples of interruptions that were detected with the algorithm displayed in 2D grayscale images (Figs 3+5). In most cases, a clear interruption in the cortex can be seen, whereas in some cases an interruption was detected which could not be seen on the 2D grayscale images, as a result of the partial volume effect (Figs 3+5). This potential error might be resolved when using the second generation HR-pQCT (HR-pQCT 2). The HR-pQCT 2 has a higher spatial resolution compared to the HR-pQCT (95 μm versus 130μm, respectively). Thus, the HR-pQCT 2 might be able to detect these thin structures, which may reduce the number of interruptions detected as a result of the partial volume effect.

However, with the HR-pQCT 2, the cortical mask thickness should be increased to 5 voxels (= 0.305mm), to obtain a similar cortical mask thickness as for the first generation HR-pQCT. In addition, the minimum diameter of interruptions is smaller on the HR-pQCT 2 due to the smaller voxel size (≥0.183mm versus ≥0.246mm). Hence, the HR-pQCT 2 is able to detect even smaller interruptions, which might lead to a higher number of interruptions detected.

The fully-automated differed from the semi-automated procedure in the detection of large cortical interruptions (diameter>1mm). In the first step of the fully-automated algorithm, where the outer contour of the bone is identified, the original outer contour of the finger joint may not be restored in the case of large interruptions. In the example of Fig 4a, both operators manually corrected this. For small cortical interruptions (diameter<1mm) no correction was necessary. The role for HR-pQCT will mainly be for the detection of small cortical interruptions, as larger interruptions are already visible on other imaging techniques. Although not specific for RA [10], the small interruptions might be prone to bone resorption early in the course of RA [23]. Clinical follow-up studies in patients with RA are needed to investigate whether these small interruptions may become established erosions.

Some limitations of the study should be mentioned. First, we included a small number of subjects, however, a large number of cortical interruptions (199 in total) was detected in these joints, which provided us reliable estimates for the proportion of matching interruptions. Second, the cortical mask was set to a constant thickness because this would avoid any inaccuracies in the detection of the endosteal cortical bone contour. A disadvantage of our approach is that in relatively thick cortical regions, that are located more distal from the joint, cortical interruptions can be detected which are not connected to the endosteal cortical bone surface, as was exemplified in Fig 4b. In addition, the interruption surface may be overestimated in thin cortical regions, because a small part of the trabecular region is considered as part of the interruption.

As RA is characterized by the development of cortical interruptions in hand joints, we expect that this fully-automated algorithm can help in the assessment of small cortical interruptions in finger joints by HR-pQCT. The algorithm needs to be further validated in clinical studies in order to obtain its potential value.

In conclusion, the algorithm presented in this study allows the detection of cortical interruptions, and its interruption surface. Reliability and validity was comparable for the fully- and semi-automated procedures. However, we advise the use of the semi-automated procedure to assure quality. We expect that this algorithm is a promising tool for a sensitive and objective assessment of cortical interruptions in finger joints by HR-pQCT.

## Supporting information

S1 Fig An illustration of the auto-contouring script. An illustration of the auto-contouring script of a 2D grayscale image with a cortical interruption. The grayscale image (a) is thresholded for a first structure approximation using Gaussian filtering (sigma = 0.8, support = 1 voxel) and a constant threshold of 105 per 1000 of maximum possible voxel value (b). This structure is dilated by 7 voxels (c). After deleting (by ranking) the black voxels inside the dilated structure (red arrow, d), the volume was eroded back to its original size (e). The contour that is obtained is displayed in green in the original grayscale image (f), and used for segmentation of the bone using the standard evaluation protocol with a constant threshold using Laplace-Hamming filtering (g).
(TIF)

S1 File MP_InterruptionDetection_246um.com;1Code of the algorithm for (semi-)automated detection of interruptions ≥0.246mm. (COM)

S2 File DATA OP1-OP2-AUTO-Visual_FINAL.sav. Data of the number of interruptions detected with the algorithm and visually.
(SAV)

S3 File AlgorithmVisual_MatchingBreaks.xlsx. Data of the number of matching interruptions of the algorithm and visual scoring. Thus, between AUTO-OP1, AUTO-OP2, OP1-OP2, and AUTO-Visual, OP1-Visual and OP2-Visual.
(XLSX)
