# Correction: An automated algorithm for the detection of cortical interruptions on high resolution peripheral quantitative computed tomography images of finger joints

**DOI:** 10.1371/journal.pone.0179138

**Published:** 2017-06-02

**Authors:** The PLOS ONE Staff

There is an error in the fourth sentence of the Results subsection of the Abstract. The correct sentence is: Also the inter-operator reliability was almost perfect (ICC≥0.97, proportion of matching interruptions 82%). The publisher apologizes for the error.
