# Supplementary figures and images for: An automated algorithm for the detection of cortical interruptions on high resolution peripheral quantitative computed tomography images of finger joints
Source: PLoS One. 2017 Apr 20;12(4):e0175829. doi: 10.1371/journal.pone.0175829 (PMC5402632; doi:10.1371/journal.pone.0175829)

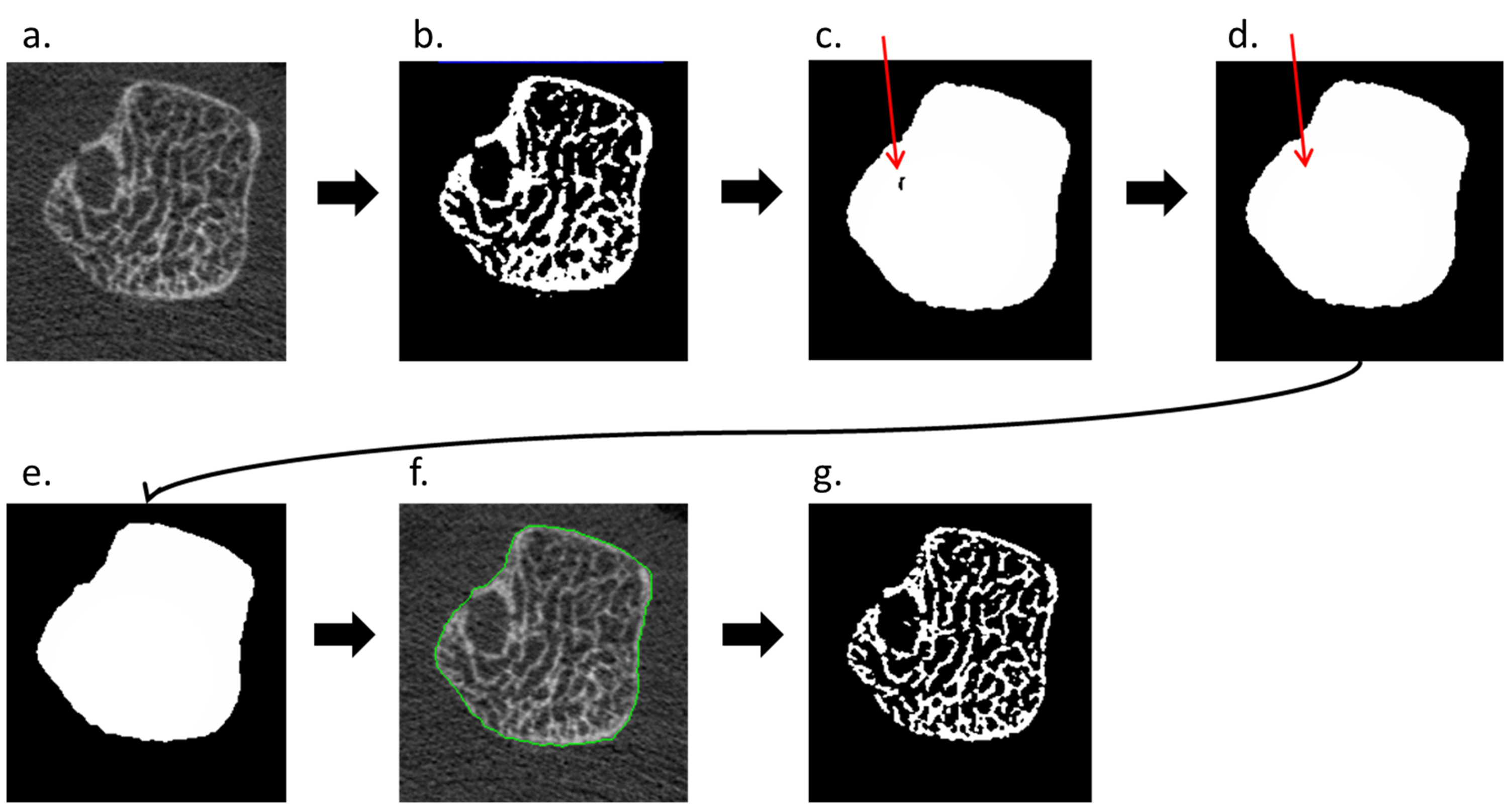

Supplement: S1 Fig — An illustration of the auto-contouring script of a 2D grayscale image with a cortical interruption. The grayscale image (a) is thresholded for a first structure approximation using Gaussian filtering (sigma = 0.8, support = 1 voxel) and a constant threshold of 105 per 1000 of maximum possible voxel value (b). This structure is dilated by 7 voxels (c). After deleting (by ranking) the black voxels inside the dilated structure (red arrow, d), the volume was eroded back to its original size (e). The contour that is obtained is displayed in green in the original grayscale image (f), and used for segmentation of the bone using the standard evaluation protocol with a constant threshold using Laplace-Hamming filtering (g). (TIF) [file pone.0175829.s001.tif]
